# The Role of Personality and Subjective Exposure Experiences in Posttraumatic Stress Disorder and Depression Symptoms among Children Following Wenchuan Earthquake

**DOI:** 10.1038/s41598-017-17440-9

**Published:** 2017-12-08

**Authors:** Xiacan Chen, Jiajun Xu, Bin Li, Na Li, Wanjun Guo, Mao-Sheng Ran, Jun Zhang, Yanchun Yang, Junmei Hu

**Affiliations:** 1 West China School of Basic Medical Sciences and Forensic Medicine, Sichuan University, Chengdu, China; 2 Mental Health Centre, West China Hospital, Sichuan University, Chengdu, China; 3 Mental Health Centre of Sichuan University, Chengdu, China; 4 Department of Social Work and Social Administration, the University of Hong Kong, Hong Kong, China

## Abstract

This study aims to investigate the role of personality traits and subjective exposure experiences in posttraumatic stress disorder and depression symptoms. In Qingchuan, 21,652 children aged 7 to 15 years were assessed using face-to-face interviews one year after the Wenchuan earthquake in China. The Junior Eysenck Personality Questionnaire, a modified earthquake exposure scale, the UCLA Posttraumatic Stress Disorder Reaction Index (adolescent), and the Adolescent Depression Inventory were used to assess personality characteristics, trauma experiences, posttraumatic stress disorder and depression symptoms, respectively. The measurement was completed with 20,749 children. After adjusting for other factors by multinomial logistic regression analysis, neuroticism, having felt unable to escape from the disaster and having been trapped for a longer time were risk factors of posttraumatic stress disorder and depression symptoms. Socialization was a protective factor of them. Having felt extreme panic or fear was a risk factor of posttraumatic stress disorder symptoms. For depression symptoms, introversion and psychoticism were risk factors, and extraversion was a protective factor. This study was conducted with the largest representative sample of child survivors of a natural, devastating disaster in a developing country. These results could be useful for planning psychological intervention strategies for children and for influencing further research.

## Introduction

On May 12, 2008, a devastating earthquake measuring 8.0 on the Richter scale struck southwestern China, with the epicentre located in the Yingxiu town of Wenchuan County, Sichuan province^1^. It was reported that 69,195 people died, 374,177 people were injured, and 18,403 people went missing^2^. These losses may have caused the onset of psychiatric disorders for the survivors. Posttraumatic stress disorder (PTSD)—a trauma- and stressor-related disease—and depression, two of the most common psychiatric disorders observed among earthquake survivors, may surface weeks or months after traumatic events and often persist for years^3^. According to the Diagnostic and Statistical Manual of Mental Disorders, fifth edition (DSM-5), the diagnostic criteria of PTSD among school-aged children (older than 6 years) and young adolescents is the same as that of adults; however, the symptomatology of PTSD presented among children and adolescents may show increased arousal and persistent negative emotional states, which differs from that of adults^4,5^.

Personality traits determine the variation in typical reactions to the environment. That is, even when individuals experience the same trauma, their responses can vary because of personality traits. These traits are one reason why many people might experience trauma, but only a few develop PTSD^6^. Individual traits affect the development of PTSD by shaping cognitive processes, coping strategies and interaction with social support processes^7–9^. In predicting PTSD, the neuroticism trait is considered the most important personality dimension as it has been found to be robustly related to many mental disorders^10^. This trait, characterised by a presentation of negative emotional responses to threat, frustration or loss, overlaps specific aspects with arousal symptoms. This content-overlap is why neuroticism is considered a vulnerability factor of PTSD^11,12^. Extraversion, characterised by being outgoing, social, talkative, and having high positive affect, is associated with social support when detects the relationship between extraversion and PTSD^13,14^. Extraversion has been addressed by many researchers as negatively connected with PTSD; however, other researchers have failed to replicate this assertion^15^.

However, the above studies have been pertinent to adults, and the majority of the studies have ignored the impact of other personality traits (e.g., psychoticism). It has not yet been determined what role personality traits play in PTSD or depression symptoms among children after a catastrophe when primary personality traits are included and other risk factors are controlled^16^.

Another critical cluster of risk factors associated with PTSD in children is trauma exposure experiences that can be divided into two categories: objective (e.g., losing a family member, injury) and subjective (e.g., the experience of intense fear, helplessness, or horror)^17,18^. The former experiences have been found in many studies to have a consistent positive relationship with the incidence of PTSD^19,20^; by contrast, the latter experiences have been reported in only a few studies^21,22^. Notably, a meta-analysis has discovered that subjective exposure experiences are high-risk factors of PTSD, where as objective experiences appear only as small- to medium-risk factors. Research has shown mixed findings about the relationship between subjective characteristics and depression^22^; therefore, research should put additional emphasis on subjective trauma characteristics^18^.

Because of high comorbidity (varying from 20 to 45%), depression symptoms are strongly inter-correlated with PTSD after catastrophes^23–26^. Depression shares some risk factors with PTSD symptoms (e.g., neuroticism, objective trauma experiences)^10,27^, but some factors still show mixed findings (e.g., extraversion)^28,29^. However, with changes in the maturation process from child to adult, these factors might lead to a different impact of depression among children, which is not often reported. Therefore, the impact of these factors on depression among children should be studied further. Compared with the experience of PTSD on its own, patients with PTSD and depression present more severe symptoms, lower executive function and worse prognosis and often require different treatment strategies^30–32^. Despite these different manifestations with the presence of comorbidity, only a few studies have focused on the predictors of comorbid PTSD and depression as a separate entity from PTSD, with even fewer studies among children^33^.

It is notable that very little research on PTSD or depression has been conducted with large samples after a catastrophe, and the baseline of stress reactions that develops naturally among children in developing countries after a catastrophe has not been applied. Moreover, some non-psychological risk factors of PTSD or depression, such as sex, age, earthquake severity and distance to the epicentre, have been previously reported by many researchers^20,24,34^. Therefore, it is necessary to control for these non-psychological confounding risk factors. Finally, better understanding of the questions mentioned above could help us screen affected children in a timelier manner and provide some useful information for psychological prevention through which we can ultimately reduce the suffering of affected children. Given these reasons, we aimed to conduct a survey among child survivors to explore the role of primary personality traits and subjective exposure experiences (under the control of other risk factors) in PTSD and depression symptoms and offer a baseline for the stress reactions of children after a disaster. We hypothesised that personality traits and subjective exposure experiences are vital risk factors of PTSD and depression symptoms, even when controlling for other risk factors. Reducing the suffering of these children is our ultimate interest. We hope to do this by identifying susceptible children, providing useful information for following up on the affected children, and providing applications for these results in clinical practice.

## Results

### Demographic characteristics

Of the 21,652 participants, 20,749 (95.8%) children finished the measurement. Table 1 presents the demographics, personality characteristics, earthquake exposure experiences, and mental health outcomes of the children. The mean and median age of the children who finished the assessment (N = 20,749) was 11.7 (SD = 2.3) and 12 (IQR: 10, 12, 14), respectively. The majority of them were of Han nationality and from rural areas. In total, the prevalence of PTSD and depression symptoms was 19.2% (95% CI: 18.7-19.7) and 31.2%, (95% CI: 30.6–31.8), respectively. The prevalence of comorbidity was 11.7% (95% CI: 11.3–12.1), and 61% of those with PTSD suffered depression one year after the earthquake.

**Table 1:** Demographic, Personality Characteristics, Earthquake exposure experiences, and Mental Health Outcomes of Children Following Wenchuan Earthquake—Qingchuan.

	Total (*N* = 20,749)	PTSD symptom (*N* = 3,982)	Depression symptom (*N* = 6,475)	PTSD comorbid depression (*N* = 2,428)
	n (%)	n (%)	n (%)	n (%)
Demographic Factors				
Boys	10338(49.8)	1826(45.9)	3153(48.7)	1114(45.9)
Age group (year) 12~15	11775(56.7)	2142(53.8)	3714(57.4)	1388(57.2)
Han Ethnic group	20323(97.9)	3888(97.6)	6325(97.7)	2376(97.9)
Residence: Town	2466(11.9)	403(10.1)	622(9.6)	237(9.8)
Removal to another area after earthquake	8474(40.8)	1710(42.9)	2865(44.2)	1077(44.4)
Personality				
P subscale				
Psychoticism	3021(14.6)	849(21.3)	1499(23.2)	598(24.6)
Moderate psychoticism	1763(8.5)	413(10.4)	745(11.5)	275(11.3)
Intermediate	10519(50.7)	2046(51.4)	3168(48.9)	1192(49.1)
Moderate socialization	3430(16.5)	465(11·7)	722(11.2)	251(10.3)
Socialization	2016(9.7)	209(5.2)	341(5.3)	112(4.6)
E subscale				
Extraversion	386(1.9)	82(2.1)	93(1.4)	41(1.7)
Moderate extraversion	1002(4.8)	176(4.4)	196(3.0)	84(3.5)
Intermediate	8595(41.4)	1505(37.8)	2141(45.3)	812(33.4)
Moderate introversion	3663(17.7)	648(16.3)	1110(17.1)	395(16.3)
Introversion	7102(34.2)	1571(39.5)	2935(45.3)	1096(45.1)
N subscale				
Neuroticism	4144(20.0)	1676(42.1)	2498(38.6)	1259(51.9)
Moderate neuroticism	2855(13.8)	735(18.5)	1261(19.5)	448(18.5)
Intermediate	9137(44.0)	1351(33.9)	2284(35.3)	658(27.1)
Moderate Stability	2656(12.8)	150(3.8)	287(4.4)	40(1.6)
Stability	1956(9.4)	70(1.8)	145(2.2)	23(0.9)
Earthquake exposure				
Saw any one dead or injured	14276(68.8)	2987(75.0)	4606(71.1)	1848(76.1)
Felt others’ panic	18041(86.9)	3595(90.3)	5739(88.6)	2213(91.1)
Trapped for a longer time	6891(33.2)	1801(45.2)	2717(42.0)	1135(46.7)
Felt one’s own or a family member’s life to be in danger	14371(69.3)	3099(77.8)	4830(74.6)	1948(80.2)
Felt unable to escape from the disaster	10694(51.5)	2683(67.4)	4082(63.0)	1713(70.6)
Felt extreme panic or fear	17143(82.6)	3623(91.0)	5581(86.2)	2199(90.6)
Lost a close family member or friend	4881(23.5)	1253(31.5)	1988(30.7)	840(34.6)
Had a close family member or friend injured	8256(39.8)	1942(48.8)	3040(46.9)	1264(52.1)
Lost home or important belongings^a^	13920(67.1)	2994(75.2)	4618(71.3)	1842(75.9)
Injured	1756(8.4)	490(12.3)	804(12.4)	343(14.1)

### Correlations of Variables

The Spearman correlation coefficients presented in Table 2 indicated that although some of these variables were correlated, the relationships were weak (γ < 0.3), except for the relationship between having a close family member or friend injured and PTSD or depression symptoms.

**Table 2:** Inter-correlation Coefficient Matrix of Demographic, Personality Characteristics, Earthquake exposure experiences and Mental Health Outcomes—Qingchuan.

	Sex	Demographic					Personality		A1	A2	Earthquake exposure experience								Symptom	
		Age	Ethic	Res	Rem	P	E	N			A3	A4	A5	A6	A7	A8	A9	A10	PTSD	Dep
Sex	1																			
Age group (Age)	0.008	1																		
Ethic group (Ethic)	−0.011	0.019**	1																	
Residence (Res)	0.013	−0.097**	0·002	1																
Removed to another area after earthquake (Rem)	0.005	−0.096**	−0·017*	0.119**	1															
Psychoticism (P)	0.026**	−0.023**	−0.004	0.020**	0.044**	1														
Extraversion (E)	0.024**	−0.011	−0.003	−0·016*	0.013	0.044**	1													
Neuroticism (N)	−0.031**	−0.005	0.012	0.015*	0.029**	0.095**	0.036**	1												
Saw any one dead or injured (A1)	−0.025**	0.138**	0.014*	0.021**	0.053**	−0.014*	−0.031**	0.028**	1											
Felt others’ panic (A2)	0.002	0.019**	−0.004	0.006	0.014*	−0.015*	−0.007	0.034**	0.063**	1										
Trapped for a longer time (A3)	−0.026**	−0.107**	−0.047**	−0.013	0.066**	0.019**	0.001	0.044**	0.130**	0.057**	1									
Felt one’s own or a family member’s life to be in danger (A4)	−0.024**	0.049**	0	−0.047**	0.031**	−0.011	−0.018**	0.057**	0.109**	0.212**	0.111**	1								
Felt unable to escape from the disaster (A5)	−0.059**	−0.082**	−0.020**	−0.029**	0.032**	0	−0.002	0.078**	0.048**	0.118**	0.159**	0.244**	1							
Felt extreme panic or fear (A6)	−0.127**	−0.065**	−0.017*	−0.058**	0.007	−0.023**	−0.017*	0.035**	0.055**	0.203**	0.088**	0.182**	0.228**	1						
Lost a close family member or friend (A7)	−0.006	−0.053**	0.01	0.023**	0.094**	0.014	−0.01	0.051**	0.171**	0.001	0.152**	0.078**	0.094**	0.007	1					
Had a close family member or friend injured (A8)	0.006	0.086**	0.017*	0.013	0.062**	0.007	−0.014*	0.060**	0.251**	0.030**	0.113**	0.108**	0.064**	0.024**	0.332**	1				
Lost home or important belongings^a^ (A9)	−0.026**	0.051**	−0.009	−0.049**	0.099**	−0.005	−0.024**	0.030**	0.198**	0.066**	0.116**	0.111**	0.098**	0.083**	0.131**	0.174**	1			
Injured (A10)	0.035**	−0.020**	0.01	0.006	0.071**	0.034**	0.01	0.042**	0.088**	−0.007	0.125**	0.040**	0.054**	0.003	0.168**	0.190**	0.063**	1		
PTSD	−0.039**	−0.029**	−0.011	−0.027**	0.021**	0.039**	0.017*	0.213**	0.065**	0.048**	0.124**	0.090**	0.154**	0.108**	0.091**	0.089**	0.084**	0.067**	1	
Depression (Dep)	−0.015*	0.008	−0.013	−0.047**	0.047**	0.095**	0.054**	0.258**	0.034**	0.034**	0.125**	0.078**	0.155**	0.063**	0.114**	0.099**	0.061**	0.096**	0.313**	1

### Risk Factors of PTSD and Depression

After controlling for confounding factors, the risk factors of only PTSD symptoms were neuroticism and three subjective exposure factors, including having felt unable to escape from the disaster, having experienced extreme panic or fear and having been trapped for a longer time. Socialization and stability were the protective factors (Table 3).

**Table 3:** Multinomial Logistic Analysis of Risk factors for Children with PTSD, Depression and PTSD co-morbid Depression Symptoms Following Wenchuan earthquake—Qingchuan.

	Only PTSD symptoms	Only Depression symptoms	PTSD co-morbid Depression symptoms
	OR (95% CI)	OR (95% CI)	OR (95% CI)
Demographic Factors			
Boys	0.92(0.83–1.03)	1.01(0.93–1.09)	0.92(0.83–1.01)
Age(year) 12–15	0.76(0.68–0.85)**	1.16(1.07–1.26)**	1.18(1.06–1.30)**
Han Ethnic group	0.73(0.52–1.02)	0.76(0.59–0.98)*	0.81(0.58–1.13)
Residence: Town	0.82(0.68–0.98)*	0.75(0.66–0.85)**	0.74(0.63–0.88)**
Removed to another area after earthquake	0.94(0.84–1.05)	1.13(1.05–1.23)**	1.04(0.94–1.15)
Personality			
P subscale			
Psychoticism	1.11(0.94–1.30)	1.63(1.46–1.81)**	1.58(1.39–1.80)**
Moderate Psychoticism	0.94(0.77–1.14)	1.29(1.13–1.47)**	1.18(1.00–1.40)*
Moderate Socialization	0.79(0.68–0.93)**	0.76(0.67–0.85)**	0.75(0.64–0.88)**
Socialization	0.64(0.52–0.81)**	0.64(0.55–0.75)**	0.62(0.50–0.77)**
E subscale			
Extraversion	1.11(0.78–1.59)	0.61(0.44–0.84)**	0.66(0.45–0.96)*
Moderate Extraversion	1.13(0.89–1.44)	0.66(0.53–0.82)**	0.80(0.61–1.04)
Moderate Introversion	0.88(0.76–1.03)	1.30(1.17–1.45)**	1.21(1.05–1.39)*
Introversion	1.09(0.95–1.23)	2.03(1.862.22)**	2.10(1.87–2.34)**
N subscale			
Neuroticism	2.72(2.36–3.13)**	3.34(3.02–3.69)**	7.95(7.06–8.94)**
Moderate Neuroticism	1.89(1.63–2.21)**	2.09(1.88–2.33)**	2.81(2.45–3.23)**
Moderate Stability	0.47(0.38–0.58)**	0.49(0.43–0.57)**	0.20(0.14–0.28)**
Stability	0.31(0.23–0.42)**	0.34(0.28–0.42)**	0.17(0.11–0.27)**
Earthquake exposure experiences			
Saw any one dead or injured	1.13(0.99–1.28)	0.89(0.81–0.97)**	1.08(0.96–1.22)
Felt others’ panic	0.97(0.82–1.16)	1.01(0.90–1.13)	1.17(0.99–1.39)
Trapped for a longer time	1.52(1.36–1.71)**	1.44(1.33–1.57)**	1.69(1.52–1.87)**
Felt one’s own or a family member’s life to be in danger	1.02(0.90–1.16)	0.98(0.90–1.07)	1.22(1.08–1.38)**
Felt unable to escape from the disaster	1.47(1.31–1.65)**	1.43(1.32–1.55)**	1.94(1.742.16)**
Felt extreme panic or fear	2.01(1.66–2.44)**	1.07(0.96–1.19)	1.57(1.34–1.85)**
Lost a close family member or friend	1.15(1.00–1.31)*	1.35(1.23–1.49)**	1.50(1.34–1.68)**
Had a close family member or friend injured	1.15(1.02–1.29)*	1.18(1.08–1.28)**	1.33(1.19–1.48)**
Lost home or important belongings^a^	1.36(1.20–1.54)**	1.09(1.00–1.19)*	1.33(1.18–1.49)**
Injured	1.15(0.94–1.40)	1.40(1.22–1.60)**	1.49(1.27–1.75)**

*P < 0.05, **P < 0.01; OR, odds ratio; CI, confidence interval.

Adjusting for confounding factors, the risk factors of only depression symptoms were neuroticism, introversion, psychoticism, and two subjective exposure experiences, including having felt unable to escape from the disaster and having been trapped for a longer time. Socialization and extraversion were the protective factors (Table 3).

After adjusting for confounding factors, children who developed PTSD with comorbid depression symptoms were more likely to have psychoticism, extraversion, and introversion traits, have been injured, and have felt that one’s own or a family member’s life was in danger. These risk factors, however, did not apply to only PTSD symptoms (Table 3).

## Discussion

This study was conducted with the largest representative sample to date of child survivors from a natural, devastating disaster in a developing country. While controlling for other risk factors, this study detected the influence of all primary personality traits and subjective exposure experiences on PTSD and depression symptoms. Before this survey, the local children had little opportunity to receive extensive and systemic psychological interventions due to their harsh natural environment and adverse traffic conditions. Accordingly, their post-earthquake stress reaction may be more likely to develop along a natural psychological process, which could in turn establish a baseline of stress reaction among children after a disaster. Since this survey was conducted one year after the earthquake, these results reflected a mid- to long-term impact of the disaster on children.

The results of this study indicated that PTSD and depression symptoms were common among children one year after the earthquake. The prevalence of PTSD has varied widely in previous reports^20^, even for the same earthquake–the Wenchuan earthquake^35^. In this study, the prevalence of PTSD symptoms (19.2%) was in line with many previous studies^36,37^. The prevalence of depression varied widely from approximately 13% to 76% after disastersworldwide^34,38–40^ and from approximately 19% to 45% after the Wenchuan earthquake^41–44^. The result of this study (31.2%) was reported to be in accordance with other research conducted after an earthquake with a similar magnitude (31%)^24^, whereas other studies reported approximately 13%^20,45^. We attributed the much lower prevalence of PTSD and depression to the lower earthquake severity (5.9 magnitude) in latter studies. The prevalence of comorbidity was high, which was in accordance with previous studies.

The neuroticism trait was a risk factor of only PTSD symptoms, and stability was its protective factor after controlling for other confounders. In the presence of neuroticism, children were 8 times more likely to suffer PTSD symptoms than those with a stability trait. Notably, in the presence of high neuroticism, the risk for children to suffer a co-occurrence of PTSD and depression symptoms was 40 times more than for children with the stability trait. These results further confirmed the findings of previous studies that neuroticism has played an important role in predicting the probability of PTSD due to its negative mood feature^46,47^. Moreover, the overlapping aspect of neuroticism and arousal symptoms might contribute to its effect on elevating the risk of PTSD^48^. Based on the results of this study, we recommend that future studies evaluate the neurotic personality as an essential risk factor for PTSD among children.

Extraversion was not associated with PTSD among children, but it was a protective factor of the comorbidity of PTSD and depression symptoms. Unlike neuroticism, extraversion was less consistent with PTSD in previous research. Some researchers revealed that extraversion had no relationship with PTSD^9,49^, whereas a negative relationship was reported in other studies^13,50^. According to the results of this study, whether the impact of comorbid depression is controlled may explain the difference, which implies the critical role of comorbid depression in detecting the impact of extraversion on PTSD. Psychoticism, reported as a risk factor in only a few earlier studies^51,52^, was rarely identified as a risk factor for PTSD in this study, unless PTSD appeared with comorbid depression symptoms. Accordingly, future studies should consider the impact of comorbid depression.

In this study, subjective exposure experiences—feeling unable to escape from the disaster, experiencing extreme panic or fear and being trapped for a longer time—were identified as risk factors for PTSD, which was similar to the findings of other researchers^53,54^. Another study showed that children who experienced extreme panic or fear had a 9-timehigher risk for PTSD symptoms than children without this experience^22^, whereas our result only showed a two-time higher risk. We believe that controlling the impact of personality traits in our study could explain the lowered risk. Inability to escape a traumatic stressor—that is, the uncontrollability of a stressor—has been found to be a crucial risk factor for PTSD in trauma survivors^55^, and a perceived threat is very likely to be linked to the onset of PTSD. In addition, research conducted on earthquake survivors has shown that fear or a sense of threat during earthquake exposure is a strong predictor of PTSD symptoms, explaining a greater proportion of variance than objective trauma severity variables, such as having been trapped under rubble or being injured^20,56–58^. The objective exposure experiences (e.g., the severity of the natural disaster or being injured) cannot be altered after the disaster, but the subjective experiences can be changed by effective psychological intervention. Therefore, future psychological interventions should focus on ameliorating children’s subjective trauma characteristics, which could, in turn, reduce the experience of PTSD symptoms. Future research should pay more attention to subjective exposure experiences.

For depression symptoms in the absence of comorbid PTSD symptoms, neuroticism could increase the risk of depression in children, consistent with the findings of earlier studies^59^. Moreover, we found that children with the neurotic trait were approximately 11 times more likely to suffer depression symptoms than those with the stability trait.

Unlike PTSD, extraversion and introversion were the protective factor and the risk factor, respectively, of only depression symptoms in this study. Lower extraversion was reported to relate to higher depression rates in adults^60,61^, which was also discovered among children in this study after controlling for other risk factors. However, a lack of this association was reported in another study of adolescents^29^. We attribute the opposite result to the different assessments used to measure extraversion. Extraversion is composed of four major facets, and some questionnaires assess only one or two primary facets^62,63^. This difference highlights the fact that various facets of extraversion may show somewhat divergent associations with depression. Therefore, in the future, research should focus on the impact of a particular facet on depression instead of the whole extraversion dimension. These outcomes regarding personality traits could be beneficial for formulating appropriate psychological interventions to lessen depression symptoms.

One subjective exposure experience—having felt unable to escape from the disaster—which was found to be a risk factor of depression symptoms in early research^64^, was replicated in this study, although other studies did not identify this association^22^. The difference in sample size and whether the research studies controlled for the other risk factors might account for the mixed findings.

This study had some limitations. First, this was a cross-sectional investigation, whereas a long-term follow-up study may be more worthwhile. Second, instead of clinician-verified diagnoses of PTSD and depression, symptoms of PTSD and depression were screened only by the instruments; however, these instruments have been widely used for research and have good reliability and validity, which guaranteed the outcomes of this study. Third, there could be some bias in recalling subjective experience a year after the disaster. However, the questionnaire was completed by children without the parent or guardian in close proximity, which might lower the bias to some degree. Finally, this research simultaneously measured children’s personality characteristics and symptoms post-earthquake, which could mean that the earthquake or symptoms themselves may have had an influence on the development of personality traits.

## Methods

### Procedure and Samples

**Figure 1:**
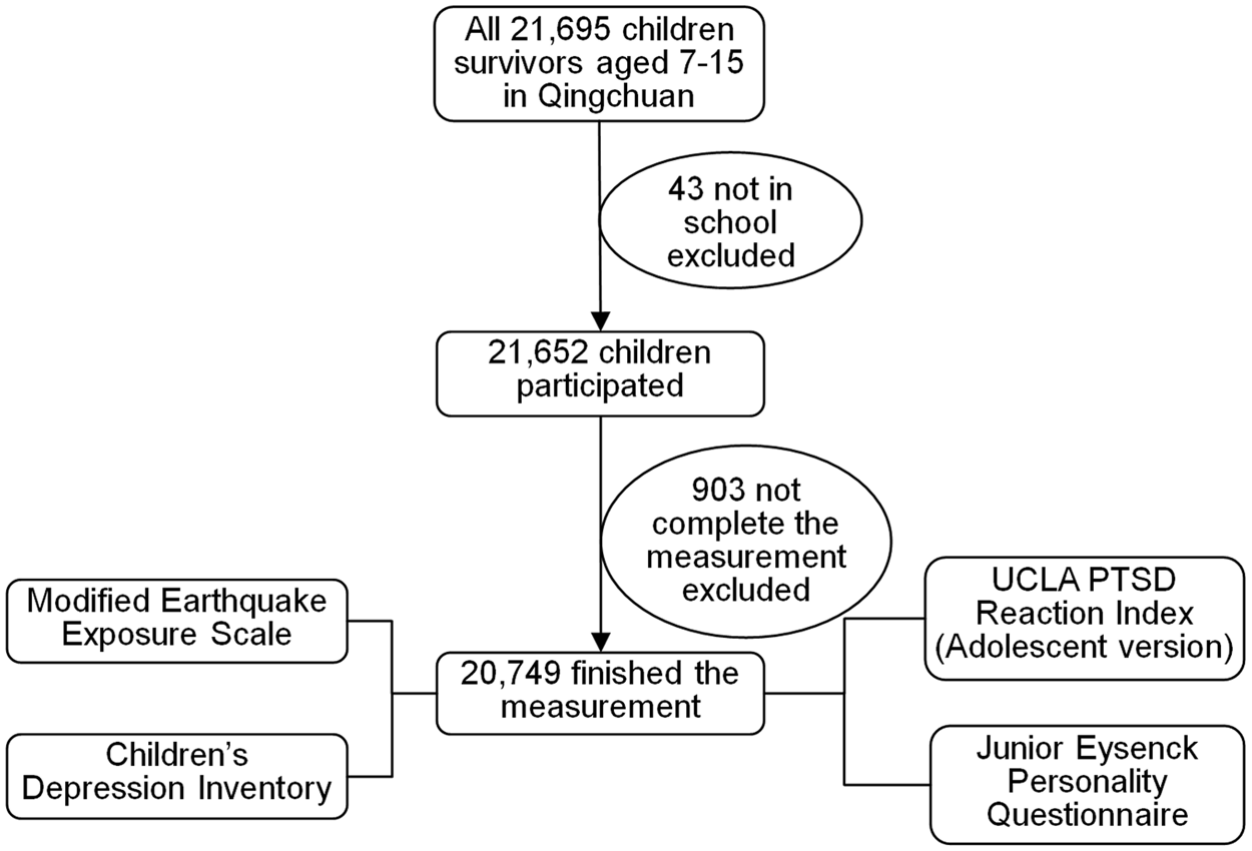
Sampling Stages for Children Survivors in Qingchuan, Sichuan Province Following the Wenchuan Earthquake.

To establish a baseline for stress reactions that develop as a result of natural processes, Qingchuan, Sichuan province, in China (a region with the 10th strongest seismic intensity and a population of 248,000 encompassing an area of 3,271 square kilometres) was selected as the survey site (Fig. 1). After the disaster, extensive and systemic psychological interventions were minimally provided for the children in Qingchuan until the survey could be implemented because of the harsh natural environment and poor traffic conditions. This ensured a naturally developing process of the children’s post-earthquake stress reaction. The participants were recruited from schools in Qingchuan approximately one year after the Wenchuan earthquake, from May to July 2009. The timing for evaluating post-earthquake stress reactions is crucial, because within one year after the disaster, the manifestations in child survivors may either vanish or become more permanent^65,66^.

There were 21,695 children aged 7–15 years old in Qingchuan. Of these children, 99.8% (21,652) of those who experienced the Wenchuan earthquake, participated this survey, and 95.6% (20,749) finished the measurement. The survey was conducted by trained interviewers through face-to-face interview. The interviewers included psychiatrists, psychologists, psychiatric nurses, and social workers. Participants were interviewed in school without the parent or guardian in close proximity; thus, each child responded to all questions without consultation or interference. All children were asked for consent, and their parents or guardians provided informed consent to participate.

This study was considered the foundation for applying further appropriate psychological interventions targeted at child survivors who were at elevated risk of developing or experiencing either PTSD or depression symptoms in Qingchuan. Children in need of mental health support were referred to available mental health services after this survey. Because of the seriousness of the psychological stress-related disorder in children post disaster, the study was conducted under the stringent supervision of the local education department. When the research was initiated, West China Hospital did not require ethics approval for research that involved secondary data analyses. To ensure compliance with ethics requirements, the present research was reviewed and received ethics approval from the Medical Ethics Committee of West China Hospital on 2 April 2015. The survey was totally performed in accordance with relevant guidelines and regulations.

### Instruments

An earthquake exposure scale, which included 10 yes-and-no questions, was used to evaluate the children. It was adapted from prior earthquake exposure scales based on the Diagnostic and Statistical Manual of Mental Disorders (4th ed., text rev.; DSM-IV-TR; American Psychiatric Association, 2000) A-1 and A-2 criteria for PTSD^67,68^ and was modified according to earthquake trauma experiences. The instrument contained five items evaluating subjective factors (i.e., having experienced extreme panic or fear, having felt unable to escape from the disaster, having felt others’ panic, having been trapped for a longer time, having felt one’s own or a family member’s life to be in danger).

The adolescent version of the UCLA PTSD Reaction Index, which is widely used to assess traumatised children following major disasters and catastrophic violence^69^, was used to evaluate symptoms of PTSD. The index contained 20 items with a 5-point scale ranging from 0 (never) to 4 (most of the time) and was translated into Chinese. A total score of 36 or higher was used to classify a child as having PTSD symptoms^70^. The internal consistency Cronbach’s α was 0.91 for the total scale, 0.89 for the factor of re-experiencing/avoidance/increased arousal symptoms, and 0.82 for numbness/pessimism symptoms, respectively^70–72^.

Symptoms of depression were assessed by the Children’s Depression Inventory (CDI), a self-report measure designed for children ages 6 to 17 years^73^. This questionnaire contained 27 items and was used to rate the frequency of depression symptoms over the previous 2 weeks on a 3-point scale (2 = most of the time,1 = sometimes, 0 = never)and then was translated into Chinese. With excellent test-retest reliability (0.75) and good internal consistency (0.82) corresponding to the DSM-IV-TR symptom groups, the questionnaire has been proven to be a valid tool for the assessment of depression symptoms in adolescents^74,75^. A score of 20 or higher was used to classify an adolescent as having depression symptoms^76^.

The Junior Eysenck Personality Questionnaire (JEPQ), a self-report questionnaire for children aged 7 to 15^77^, was translated into Chinese and modified 30 years ago^78,79^. It was used to assess personality characteristics in this study, whereas most previous research has included only one or two subscales. Since the Chinese norm was compiled, the JEPQ has been widely used in China^80,81^. The Chinese version of the JEPQ comprises88 yes-and-no items (yes = 1, no = 0) and five levels of four subscales: psychoticism (P), extraversion (E), neuroticism (N), and lie (L). Its test-retest reliability was 0.65, 0.86, 0.72 and 0.62 for the P, E, N and L subscales, respectively. For example, the E subscale was divided into five levels: introversion (T < 38.5); moderate introversion (38.5 ≤ T < 43.3); intermediate extraversion(43.3 ≤ T ≤ 56.7); moderate extraversion (56.7 < T ≤ 61.5); and extraversion (T > 61.5). The other subscales were divided similarly.

### Statistical analyses

Statistical analyses were performed by the Statistical Product and Service Solutions 16.0 (SPSS Version 16.0). First, frequencies of all variables and prevalence were calculated. The age variable was described by the median and the quartile, and divided into two groups (7- to 11-year-olds and 12- to 15-year-olds). Second, spearman correlation was used to assess the potential inter-variable associations of all variables. Finally, multinomial logistic regression analysis was used to identify the role of personality traits and subjective exposure experiences under the control of other factors. The dependent variable was divided into four groups: (a) without PTSD or depression symptoms; (b) only PTSD symptoms; (c) only depression symptoms; and d) PTSD comorbid depression symptoms. Group a) was used as a reference category for the dependent variable. This procedure yielded odd ratios with 95% confidence intervals for each variable. All the demographics, personality traits, and earthquake exposure experience factors were entered into the equation as independent factors. Female, national minority, younger age (7–11 years), living in a rural area, and the intermediate type of each personality dimension was served as the reference categories for gender, ethnic group, age, residence, and personality variables, respectively. The answer “no” was served as the reference category for the other dichotomous variables. All tests were two-tailed, and those that had p values of 0.05 or lower were treated as statistically significant in all data analyses.

### Data availability statement

The use of data will be confined to the study group, but potential collaborators or requests for data can be submitted to the corresponding author.
